# Evidence for infection but not transmission of Zika virus by *Aedes albopictus* (Diptera: Culicidae) from Spain

**DOI:** 10.1186/s13071-019-3467-y

**Published:** 2019-05-03

**Authors:** Luis M. Hernández-Triana, Elsa Barrero, Sarah Delacour-Estrella, Ignacio Ruiz-Arrondo, Javier Lucientes, Maria del Mar Fernández de Marco, Leigh Thorne, Sarah Lumley, Nicholas Johnson, Karen L. Mansfield, Anthony R. Fooks

**Affiliations:** 1 Wildlife Zoonoses and Vector-borne Diseases Research Group, Animal and Plant Health Agency, Woodham Lane, New Haw, Addlestone, Surrey KT15 3NB UK; 2 Department of Animal Pathology, Faculty of Veterinary Medicine, University of Zaragoza, Zaragoza, Spain; 3 Center for Rickettsiosis and Vector-Borne Diseases Group, Hospital Universitario San Pedro-CIBIR, Logroño, Spain; 4 Public Health England, Porton Down, Salisbury, SP4 0JG UK; 5 Faculty of Health and Medicine, University of Surrey, Guildford, Surrey GU27XH UK; 6 Department of Clinical Infection, Microbiology and Immunology, Institute of Infection and Global Health, University of Liverpool, Liverpool, UK

**Keywords:** *Aedes aegypti*, *Aedes albopictus*, Zika virus, Vector competence, Spain

## Abstract

**Background:**

A number of mosquito-borne viruses such as dengue virus (DENV), Usutu virus (USUV), West Nile virus (WNV) are autochthonously transmitted in Europe and six invasive mosquito species have been detected in this temperate region. This has increased the risk for the emergence of further mosquito-borne diseases. However, there is a paucity of information on whether European populations of invasive mosquito species are competent to transmit arboviruses. In this study, the susceptibility of *Aedes albopictus* originating from Spain and a laboratory-adapted colony of *Aedes aegypti*, was assessed for infection with, and transmission of Zika virus (ZIKV). Vertical transmission in both species was also assessed.

**Methods:**

*Aedes albopictus* colonised from eggs collected in Spain and an existing colony of *Ae. aegypti* were fed infectious blood meals containing ZIKV (Polynesian strain) at 1.6 × 10^7^ PFU/ml. Blood-fed mosquitoes were separated and maintained at 20 °C or 25 °C. Legs, saliva and bodies were sampled from specimens at 7, 14 and 21 days post-infection (dpi) in order to determine infection, dissemination and transmission rates. All samples were analysed by real-time RT-PCR using primers targeting the ZIKV NS1 gene.

**Results:**

At 14 dpi and 21 dpi, ZIKV RNA was detected in the bodies of both species at both temperatures. However, live virus only was detected in the saliva of *Ae. aegypti* at 25 °C with a transmission rate of 44%. No evidence for virus expectoration was obtained for *Ae. albopictus* under any condition. Notably, ZIKV RNA was not detectable in the saliva of *Ae. aegypti* at 20 °C after 21 days. No vertical transmission of ZIKV was detected in this study.

**Conclusions:**

Experimental infection of *Ae. albopictus* colonized from Spain with ZIKV did not result in expectoration of virus in saliva in contrast to results for *Ae. aegypti*. No evidence of vertical transmission of virus was observed in this study. This suggests that this strain of *Ae. albopictus* is not competent for ZIKV transmission under the conditions tested.

## Background

ZIKV (*Flaviviridae*, *Flavivirus*) is an emergent mosquito-borne disease, with a natural transmission cycle involving mainly *Aedes* species and monkeys [1, 2]. The virus was first isolated from a rhesus macaque monkey (*Macaca mulatta*) and *Aedes africanus* mosquitoes in the Zika forest of Uganda in 1947 [3]. Humans are susceptible to infection with clinical symptoms varying from asymptomatic to an influenza or dengue-like syndrome associated with fever, headache, malaise and maculopapular rash [4]. However, epidemiological investigations of the recent outbreak in Brazil and earlier outbreaks in Asia and Polynesia has linked ZIKV infection to clinical manifestations such as neonatal microcephaly and increased neurological disorders such as the Guillain-Barré Syndrome [5]. This prompted the World Health Organization (WHO) to declare the ZIKV epidemic a Public Health Emergency of International Concern in 2016 [6, 7].

*Aedes aegypti* is considered the primary vector of ZIKV and *Ae. albopictus* the secondary vector. However, this is not directly reflected by the results in vector competence studies in the literature, with some studies showing that *Ae. albopictus* can be an efficient vector of ZIKV, especially at higher temperatures, with the species being able to vertically transmit the virus to their progeny [8]. In addition to ZIKV, *Ae. aegypti* and *Ae. albopictus* can transmit many other arboviruses such as DENV, chikungunya virus (CHIKV), Mayaro virus (MAYV), yellow fever virus (YFV) and WNV [9–11].

*Aedes albopictus* is common in tropical and subtropical regions of the world [12], but has managed to establish in temperate regions due to human activity such as the importation of used tyres and translocation of adult mosquitoes in cars [13, 14]. In Europe, the species is widespread throughout the Mediterranean Basin, and it is currently expanding its range to northern latitudes [15, 16]. In Spain, *Ae. albopictus* is commonly detected in coastal areas, but has colonized much cooler areas of northern Spain such as Aragón [17, 18]. Due to the potential public health risk of ZIKV being introduced into Spain and other countries in Europe [19], this study has evaluated the vector competence of a Spanish population of *Ae. albopictus* for the Asian genotype of ZIKV at two temperatures. In parallel, *Ae. aegypti* was used as a control vector with known competence for ZIKV. Furthermore, we have also assessed the potential of these two mosquito species to transmit ZIKV vertically at both temperatures.

## Methods

### Colonization of mosquitoes

*Aedes albopictus* eggs were collected in ovitraps in 2009 at Baix Llobregat municipality, Barcelona province, Spain, and a colony was established at the Faculty of Veterinary Medicine, University of Zaragoza in the same year [20]. *Aedes aegypti* (Biogents, http://www.eu.biogents.com) supplied by Dr Andrea Drago and originally colonised from mosquitoes from Cuba (year of colonization unknown), was used for comparison. Eggs were then sent to the Animal and Plant Health Agency, United Kingdom, and a colony was established within the high biosecurity containment facility at the agency in 2017. For the maintenance of mosquitoes, we followed the protocols of Alarcón-Elbal et al. [20], Hernández-Triana et al. [21] and Puggioli et al. [22].

### Cells and virus

ZIKV (strain H/PPF/2013 isolated in French Polynesia; GenBank: KJ776791) was obtained through the EVAg consortium (https://www.european-virus-archive.com). This virus is closely related to that currently circulating in South America [23]. The strain was grown and passaged at 37 °C in Vero cells. The strain was titrated in Vero cells following the protocol of Hernández-Triana et al. [21].

### Assessment of vector competence and vertical transmission

Three to five day old adult female *Ae. albopictus* or *Ae. aegypti* (number of generations from the seed colony unknown) were allowed to feed with a Hemotek feeding system (Hemotek Ltd, Blackburn, UK) at room temperature for a maximum of 2 h on an infectious blood meal containing defibrinated horse blood, adenosine 5′-triphosphate (final concentration 20 mM) and ZIKV (at 1.6 × 10^7^ PFU/ml). Mosquito infection, immobilization, processing of specimens at different days post-infection (dpi), and assessment of vector competence (infection, dissemination and transmission rates) was achieved through published protocols [21]. Infection rate was calculated by dividing the number of ZIKV RT-PCR positive mosquito bodies (abdomen/thorax) by the total number of blood-fed individuals. Dissemination rate was calculated by dividing as the number ZIKV RT-PCR positive legs by the number of positive bodies. Transmission rate was calculated by dividing the number of ZIKV RT-PCR positive saliva expectorate samples by the number of positive leg samples (Table 1).

**Table 1:** ZIKV infection, dissemination and transmission of challenged *Ae. albopictus* Spain and *Ae. aegypti* Biogens with ZIKV at 20 °C and 25 °C

		20 °C			25 °C		
		Dpi 7 (%)	Dpi 14 (%)	Dpi 21 (%)	Dpi 7 (%)	Dpi 14 (%)	Dpi 21 (%)
*Ae. albopictus*	Infection (Body)	4/9 (45%)	5/16 (31.2%)	2/15 (13.3%)	7/7 (100%)	6/10 (60%)	0/16
	Dissemination (Legs)	0/4	0/5	0/2	4/7 (54%)	0/6	0
	Transmission (Saliva)	0	0	0	0/4	0	0
*Ae. aegypti*	Infection (Body)	11/25 (44%)	12/30 (40%)	4/26 (15.3%)	12/22 (55%)	15/23 (65.2%)	12/18 (67%)
	Dissemination (Legs)	7/11 (63%)	7/12 (58%)	0/4	5/12 (41%)	12/15 (80%)	9/12 (75%)
	Transmission (Saliva)	5/7 (71%)	3/7 (42%)	0	5/5 (100%)	5/12 (41%)	5/9 (55%)

*Abbreviation*: dpi, days pos-infection

For the assessment of vertical transmission, three days after the first ZIKV infectious bloodmeal, a small plastic bowl containing water and filter paper was placed inside Insect Bugdorm cages (http://www.bugzarre.co.uk) for oviposition. The filter paper was maintained for 7 days for completion of the first gonotrophic cycle at dpi 7, after which they were collected and dried down. At this point, non-infectious blood was offered to mosquitoes and the same process was repeated twice for completion of the second (dpi 14) and third (dpi 21) gonotrophic cycles. Eggs were pooled by species and temperature, and placed to hatch and maintained in the insectary following similar published protocols [21] in order obtain F1 progeny, with the exception that eggs collected in filter papers embedded in water contained in transparent plastic bowls. Cohorts of larvae, pupae, males and females were reared and pooled prior to testing for ZIKV as detailed in Table 2.

**Table 2:** Number of pools and specimens per life stage and temperature from the F1 of *Ae. albopictus* Spain and *Ae. aegypti* Biogen tested by qRT-PCR assay to detect ZIKV Asian genotype

Species/Temperature	Life stage	No. of pools
*Aedes albopictus*/20 °C	Larvae	na
	Pupae	na
	Males	1
	Females	1
*Aedes albopictus*/25 °C	Larvae	2
	Pupae	3
	Males	4
	Females	9
*Aedes aegypti*/20 °C	Larvae	2
	Pupae	2
	Males	1
	Females	1
*Aedes aegypti*/25 °C	Larvae	19
	Pupae	8
	Males	4
	Females	11

### Processing of samples for reverse transcription (RT) PCR

RNA from larvae, pupae, legs/wings, saliva and body samples of *Ae. albopictus* and *Ae. aegypti* was extracted using TRIzol (http://www.tools.thermofisher.com) as previously described in Hernández-Triana et al. [21]. Briefly, specimens were homogenized by adding a 5 mm metallic bead to each tube and using a Qiagen Tissue Lyser (model Retsch MM301) with a tissue disruption speed of 25 MHz for 3 min. All tubes were then centrifuged for 5 min at 14,000× *rpm* and 30 μl of supernatant was collected for virus isolation assays; the RNA pellet was eluted in 20 μl of nuclease-free water. ZIKV RNA was detected using a semi-quantitative reverse transcription (RT)-PCR assay targeting a 213 nucleotide region of the NS1 gene using a newly designed primers ZIKVENV-Forward: 5′-AAG CAY TGG TTG GTK CAC A-3′; ZIKVENV-Reverse: 5′-CAC CAT CCA TCT CAG CCT-3′; ZIKENV-Probe: [6FAM]AAC TCC ACA YTG GA ACA ACA A[TAM]. The RT-PCR was performed using iTaq™ Universal Probes One-Step Kit (Bio-Rad) using an MxPro 3005P thermal cycler (Stratagene) in a 25 µl reaction mix: RNase-free water (7 µl); 2× iTaq universal probes reaction mix (12 µl); 1 µl of each primer and probe at final concentration of 10 pmol/µl; and 1 µl of iScript RT Mix Enzyme for One Step, and 2 µl of template RNA. The conditions were as follows: Reverse transcription 50 °C for 10 min; reverse transcriptase inactivation 95 °C for 5 min; and PCR amplification and detection 40 cycles consisting of 95 °C for 10 s, 55 °C for 30 s.

### Virus isolation and titration

Plaque assays were performed following the protocol of [20] on infectious pre- and post-feed blood samples to confirm the titre of virus provided, and also on representative saliva and abdomen samples which were ZIKV positive by PCR. A ten-fold dilution series of supernatant of the homogenized body parts or salivary secretions (30 μl) was prepared, and dispensed onto a confluent monolayer of Vero cells on a 12-well plate (Costar®, Corning Life Sciences, Massachusetts, USA). The plates were incubated at 37 °C in an atmosphere of 5% CO_2_ for 3 h. After incubation, 1–1.5 ml overlay of warmed 3% carboxymethylcellulose (CMC) in 250 ml 2× EMEM mix (Deionised water, 7.5% sodium bicarbonate, HEPES 1M, l-glutamine 200 mM, FBS, antibiotics (penicillin and streptomycin), and 10× MEME-E) were added to each well. After 7 days incubation, 1 ml of 10% neutral buffered formalin solution was added to each well and the plates left for least 3 h to complete virus inactivation. Wells were stained with 200 μl of 2.3% crystal violet solution.

## Results

Two *Aedes* species, *Ae. albopictus* and *Ae. aegypti*, were orally infected to investigate their vector competence for an Asian strain of ZIKV. Under our experimental conditions, female *Ae. albopictus* fed poorly, therefore the results from four experiments were combined in order to obtain sufficient numbers of infected mosquitoes for downstream analysis. In total 300 female *Ae. aegypti* and 700 female *Ae. albopictus* were offered an infectious bloodmeal containing ZIKV, resulting in feeding rates of 61% and 24%, respectively.

ZIKV nucleic acid was detected by RT-PCR in body samples of both species at 20 °C and 25 °C; however, percentages of RNA detection varied for each species. In *Ae. albopictus* at 20 °C, ZIKV RNA was detected in 45% of samples at dpi 7, 31% of samples at 14 dpi, and 13.35% of samples at 21 dpi. Conversely, at a temperature of 25 °C, infection was only detected at dpi 7 (100%) and dpi 14 (60%) (Table 1). Dissemination was only encountered at dpi 7 at 25 °C; no ZIKV RNA was detected in the saliva of *Ae. albopictus* mosquitoes at any time point. *Aedes aegypti* showed higher infection rates at both temperatures and across all dpi (Table 1). At 20 °C, dissemination rates ranged from 63% at dpi 7 and 58% at dpi 14; no dissemination was detected at dpi 21. Contrarily, at 25 °C, dissemination was detected at all dpi ranging from 41% (dpi 7), 80% (dpi 14) and 75% (dpi 21). For both temperatures, ZIKV RNA was detected in the saliva in this species (Table 1), except at 20 °C/dpi 21. Nonetheless, slightly higher transmission rates were seen at 25 °C/dpi 7, dpi 14 and dpi 21 compared to those obtained at 20 °C (dpi 7, dpi 14 and dpi 21). The presence of ZIKV in *Ae. aegypti* saliva was confirmed by plaque assay with titres ranging from 3.7 × 10^4^ to 7.4 – 10^6^ PFU/ml.

Blood-fed females of *Ae. aegypti* and *Ae. albopictus* were allowed to breed and oviposit, and the F1 generation of all different life stages (larva, pupa, adults) after ZIKV-infectious blood were sampled. Pools for both species were tested for the presence of ZIKV RNA. All pools tested from both species were negative (Table 2).

## Discussion

Due to its abundance, anthropophilic biting behaviour and ability to vector numerous arboviruses, the presence of *Ae. albopictus* represents a clear risk for the transmission of viruses such as ZIKV, especially if viraemic humans travel into infested areas. The global spread of ZIKV has in recent years dramatically increased the risk of viraemic individuals entering Europe [24] and thus encountering vectors. However, studies to assess different populations of *Ae. albopictus* have revealed varying levels of vector competence [2, 25, 26]. *Aedes albopictus* in Spain has shown a rapid increase in its distribution range in recent years, which has been facilitated in a large part by passive transportation in cars [13]. In order to assess the capacity of this population to transmit ZIKV, colonised *Ae. albopictus* from the Baix Lobregat municipality in northern Spain were orally fed a blood meal containing ZIKV and assessed for their ability to become infected, expectorate virus (a surrogate measure of bite transmission) and vertically transmit virus at 20 °C and 25 °C, temperatures that are consistently reached in northern Spain during the summer months. Infection did take place at both temperatures with an increase in efficiency at the higher temperature (Table 1). However, there was little virus dissemination and no expectoration in saliva for *Ae. albopictus* in direct contrast to the observations for the established vector for ZIKV, *Ae. aegypti.* This corroborates similar finding for *Ae. albopictus* populations in Germany [27] and Italy [24].

However, an increase in incubation temperature at or greater than 27 °C has been shown to increase the infection and transmission rates of ZIKV by *Ae. albopicutus* [5, 28, 29]. This was also demonstrated in a recent paper by Gutiérrez-López et al. [30] from *Ae. albopictus* F2 adults obtained from wild-caught eggs in Barcelona, Spain. In contrast to our results, these authors found that this population of *Ae. albopictus* was a vector of ZIKV and showed high levels of vertical transmission, which could be explained by variation in within-species population vector competence, the different virus strains and temperature used [31, 32]. A similar temperature effect has been shown for ZIKV transmission by German populations of *Ae. japonicus* [31]. In general, all in all, it seems that temperature might play be an important role for the vector competence in *Ae. albopictus*, and other *Aedes* species as discussed by Tesla et al. [33].

The relatively small number of specimens tested during experiments of ZIKV vertically transmission was due to the low number of emerged larvae and adults (Table 2). Nonetheless, the analysis of F1 offspring of both species showed no evidence of vertical transmission of ZIKV (Table 2). A similar observation has been made for *Ae. albopictus* from Italy [23], although experiments with a north American strain of the species did detect ZIKV in 1 of 84 first generation larvae, implying that vertical transmission is possible [8]. At present, few studies exist that have intensively investigated the efficiency of transovarial (vertical) and venereal (horizontal) transmission of ZIKV either in *Ae. albopictus* or *Ae. aegypti*. However, it is still possible that these forms of transmission occur at low levels to support ZIKV persistence.

## Conclusions

The colonised line of *Ae. albopictus* from northern Spain demonstrated infection with ZIKV and dissemination; however, no evidence for expectoration in saliva was detected suggesting that this strain is not a competent vector for ZIKV at the conditions assessed in this study. This is in contrast to the established vector of the virus *Ae. aegypti*. No evidence of vertical transmission in either species was detected at 20 °C or 25 °C in this study.
